# Node Deployment Algorithm for Underwater Sensor Networks Based on Connected Dominating Set

**DOI:** 10.3390/s16030388

**Published:** 2016-03-17

**Authors:** Peng Jiang, Jun Liu, Feng Wu, Jianzhong Wang, Anke Xue

**Affiliations:** 1 Key Lab for IOT and Information Fusion Technology of Zhejiang, 310018 Hangzhou, China; liujunhdu@163.com (J.L.); fengwu@hdu.edu.cn (F.W.); wangjz@hdu.edu.cn (J.W.); akxue@hdu.edu.cn (A.X.); 2 College of Automation, Hangzhou Dianzi University, 310018 Hangzhou, China

**Keywords:** underwater sensor networks, node deployment, connected dominating set, full connectivity

## Abstract

Existing node deployment algorithms for underwater sensor networks are nearly unable to improve the network coverage rate under the premise of ensuring the full network connectivity and do not optimize the communication and move energy consumption during the deployment. Hence, a node deployment algorithm based on connected dominating set (CDS) is proposed. After randomly sowing the nodes in 3D monitoring underwater space, disconnected nodes move to the sink node until the network achieves full connectivity. The sink node then performs centralized optimization to determine the CDS and adjusts the locations of dominated nodes. Simulation results show that the proposed algorithm can achieve a high coverage rate while ensuring full connectivity and decreases the communication and movement energy consumption during deployment.

## 1. Introduction

With the rapid advances in the technologies such as sensors, micro-electro-mechanical-systems (MEMS), wireless communication and embedded systems, the wireless sensor networks (WSNs) have been widely used in the applications like environment monitoring, military surveillance, industry or agriculture production, traffic control and heath care [1,2]. As the application extension of the conventional terrestrial WSNs, the underwater wireless sensor networks (UWSNs) consisting of nodes capable of sensing underwater information, processing information data, and communicating through underwater acoustic signals are a kind of monitoring system having the characteristics of acoustic signals as communication medium, 3D network structure, and limited node energy that is difficult to compensate [3,4,5].

When UWSNs are applied to detect underwater disaster or underwater military actions or to finish other similar tasks, the system must have excellent real-time monitoring, and all the nodes must be able to communicate with the sink node to guarantee full network connectivity [6]. Optimal deployment for UWSNs involves adjusting the locations of the nodes to improve network coverage and minimize energy consumption during the process, while fully considering the characteristics and requirements mentioned above. Based on the assumption for node mobility, the deployment algorithms for UWSNs can be divided into three categories [7], namely, static deployment, limited mobility deployment, and free mobility deployment. For static deployment, nodes cannot move after being manually deployed in the predetermined locations [8]. For limited mobility deployment, nodes can move in-depth and adjust their depth if necessary [9]. For free mobility deployment, nodes can freely move in all directions [10].

In recent years, some scholars studied the deployment algorithms for UWSNs based on free mobility deployment. Xia *et al.* [11,12] proposed the fish-inspired or particle swarm inspired UWSNs deployment algorithms. By simulating the behaviors of fish or particles and introducing the crowd control, the proposed algorithms could drive nodes to cover the events and match the distribution of sensors with that of events. However, this kind of algorithm only considered the network coverage rate and ignored the network connectivity rate. Liu *et al.* [13] studied the redeployment problem of nodes moving in the way of 3D random walks. In their work, the mobility of nodes caused by the water during the operation of UWSNs was considered and described using the 3D random walk model. They proposed two methods, *i.e.*, adding new nodes and moving redundant nodes to improve the network coverage rate. However, this kind of algorithm also did not consider the network connectivity rate. Li *et al.* [14] proposed the 3D virtual forces deployment (TVFD) algorithm. They modified the traditional 2D virtual forces algorithm [15] and applied it to the 3D case. The 3D algorithm proposal could be applied to the underwater 3D environment and improve the network coverage rate, as well as the network connectivity rate. However, this kind of algorithm could not guarantee full network connectivity by having network connectivity rate of 1, although this rate could be largely improved. Furthermore, all the above-mentioned algorithms ignore the optimization of communication and movement energy consumption. The energy of nodes is limited, and nodes are difficult to recharge. Thus, premature death of the node ensues, thereby affecting network lifetime.

As mentioned above, existing deployment algorithms for UWSNs consisting of freely mobile nodes cannot improve the network coverage rate under the premise of ensuring the full network connectivity and do not optimize the communication and movement energy consumption during deployment. Therefore, we propose a node deployment algorithm based on CDS (DBCDS). After nodes are randomly scattered in the 3D monitoring underwater space, nodes disconnected to the sink node are required to move toward the sink node until full network connectivity is achieved. The sink node then performs centralized optimization to determine the CDS of the network and continuously adjusts the locations of the dominated nodes. The network coverage rate can be improved using this method based on full network connectivity.

Compared with the existing deployment algorithms, the contributions of this work are as follows:

(1) The proposed algorithm is based on the CDS, and this can improve the network coverage rate under the premise of ensuring full network connectivity.

(2) The proposed algorithm can decrease the number of communication times and the movement distance during deployment, thereby optimizing the communication and movement energy consumption during deployment.

The rest of the study is organized as follows: in Section 2, the related works about the node deployment problem in the WSNs and UWSNs are elaborated. In Section 3, the models, definitions and preliminaries involved in the DBCDS algorithm are formally defined. In Section 4, the problem studied and the corresponding DBCDS algorithm are presented in detail. Section 5 provides the simulation evaluation, and Section 6 concludes the study.

## 2. Related Works

As the prime and key aspect in the WSNs design, the node deployment is not only closely related to the network monitoring quality, but also influences the follow-up protocols and algorithms such as node localization, network routing, and time synchronization [16]. For the WSNs node deployment problem, the existing algorithms can be classified into two categories: random deployment and deterministic deployment, which has attracted much attention because of its better monitoring quality compared with the former. The deterministic deployment algorithms can further be classified into four kinds based on the following mathematical approaches: computational geometry (CG), artificial potential field (APF), genetic algorithms (GA), and particle swarm optimization (PSO) [17]. Wu *et al.* [18] proposed a centralized and deterministic node deployment based on the DT-Score (Delaunary Triangulation-Score), aiming at maximizing the coverage of a given sensing area with obstacles. Zou *et al.* [15] proposed the virtual forces (VF) algorithm to improve the 2D network coverage rate after the initial node deployment, where a combination of attractive and repulsive forces were used to determine virtual motion paths and the rate of movement for the randomly-placed sensors. Jourdan *et al.* [19] proposed the multi-objective genetic (MOG) algorithm for the given number of sensors optimal deployment in the 2D flat region, aiming at maximizing the area coverage and the network lifetime. Kukunuru *et al.* [20] proposed a node deployment algorithm based on the particle swarm optimization, and the purpose of their work is to achieve the maximum coverage with the minimum number of nodes on the 2D area by minimizing the distance between the neighboring nodes.

However, owing to the differences of application environment and demands, the UWSNs have many different characteristics with the conventional terrestrial WSNs. Therefore, the node deployment algorithms for the conventional terrestrial WSNs cannot be applied directly to the UWSNs. Many researchers try to design the proper node deployment for the UWSNs. As is mentioned in Section 1, the node deployment algorithms for UWSNs can be classified into static deployment, limited mobility deployment, and free mobility deployment. For static deployment, Pompili *et al.* [21] proposed 2D and 3D UWSN structures, and performed a deep mathematical analysis on node deployment on the basis of these two structures. Besides, they studied the robustness of the sensor network to node failures, while providing an estimate of the number of required redundant sensors. Liu [22] proposed a UWSN node deployment algorithm by determining and selecting the best cluster shape after partitioning the monitored region into layers and clusters. In order to save the energy consumption and prolong the network lifetime, only the cluster-head in every cluster is kept alive for sensing and communicating. Once the cluster-head depleted its energy, a new cluster-head within the cluster would wake up and replace the old one. However, there was no description about how to keep time synchronization when waking up a new cluster-head to maintain full network coverage and connectivity. For limited mobility deployment, Akkaya *et al.* [23] proposed a distributed self-organized node deployment algorithm, which reduced overlapped coverage by adopting the graph coloring idea. Nevertheless, this algorithm emphasized on increasing network coverage rate and neglected network connectivity rate improvement. Hence, Senel *et al.* [24] put forward another distributed self-organized node deployment algorithm. It considered how to improve the network connectivity rate and maximized the network coverage rate under the premise of full network connectivity. For free mobility deployment, as has been mentioned in Section 1, existing deployment algorithms such as the proposed works in [11,12,13,14] cannot improve the network coverage rate under the premise of ensuring the full network connectivity and do not optimize the communication and movement energy consumption during deployment. Therefore, we propose the DBCDS algorithm.

As a research team which has been keeping the focus on the WSNs, we have also proposed many node deployment algorithms for the UWSNs in recent years [25,26,27,28]. The node non-uniform deployment based on clustering (NNDBC) algorithm for the UWSNs was proposed in [25], and the difference between the NNDBC algorithm and our other works in [26,27,28] as well as in this paper was that the coverage targets in NNDBC algorithm were the isolated events whose distributions were usually non-uniform in the monitored space. The algorithms in [26,27] belonged to limited mobility deployment, while the DBCDS algorithm in this paper belonged to free mobility deployment. The algorithm in [28] also belonged to free mobility deployment, the node destination locations had been calculated before the random node scattering, and the network topology formed after the algorithm operation had no relationship with the random node scattering. However, the focus of this paper is how to adjust the nodes’ locations after the random node scattering and depth adjustment, with the purpose of achieving desired performances. The node destination locations cannot be known before the random node scattering, the network topology formed after the algorithm operation has a strong relationship with the random node scattering and depth adjustment.

## 3. Models, Definitions, and Preliminaries

### 3.1. Models 

(1) 3D underwater space model

**Figure 1:**
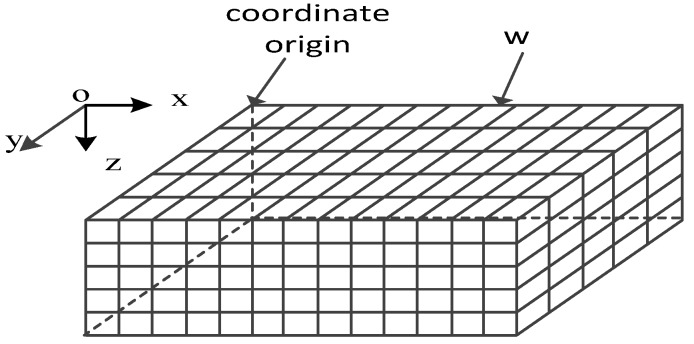
Coordinate system for UWSNs.

As is shown in Figure 1, the 3D underwater space is a large cube divided into a number of small cubes [9] whose side length is *w*, which is also called the cube resolution. All the small cubes have selected center points to represent themselves, and the coordinate of cube $p_{i}$ is $\left(a_{i},b_{i},c_{i}\right)$.

(2) Node energy consumption model

Considering that the energy consumption of nodes for sensing, processing, and receiving information is much smaller than for transmitting information and moving [29], only the latter is considered. The energy consumption for transmitting information is modelled based on the methods mentioned in [30]. Supposing that $p_{r}$ denotes the power threshold for a node to receive the information package, and d denotes the transmitting distance of the information package, the energy consumption for transmitting information is denoted as $E_{tx}\left(d\right)$, which can be calculated using the following formula:

(1) $$E_{tx}\left(d\right)=P_{r}\times T_{p}\times A\left(d\right)$$

where $T_{p}$ denotes the transmitting time of the information package and can be calculated as:

(2) $$T_{p}=\frac{M_{b}}{S_{v}}$$

where $M_{b}$ is the size of the information package, and $S_{v}$ is the transmission speed of the information package. A(d) denotes energy attenuation when the transmitting distance of the information package is d and can be calculated as:

(3) $$A\left(d\right)=d^{\lambda }\times \beta^{d}$$

where λ is the energy spreading factor (λ is 1 for cylindrical, 1.5 for practical, and 2 for spherical spreading). The parameter $\beta =10^{\alpha \left(f\right)/10}$ is determined by the absorption coefficient α(f), which can be calculated using the following formula:

(4) $$\alpha \left(f\right)=0.11\frac{10^{-3}f^{2}}{1+f^{2}}+44\frac{10^{-3}f^{2}}{4100+f^{2}}+2.75\times 10^{-7}f^{2}+3\times 10^{-6}$$

where f is the frequency of the carrier acoustic signal in KHZ, and α(f) is in dB/m. Supposing that the number of information package transmitting times for the node s is $t_{n}$, and the communication range for the node s is $R_{t}$, as well as considering the energy consumption model for transmitting information described in Equation (1), the communication energy consumption $C_{e}$ can be obtained as follows:

(5) $$C_{e}=E_{tx}\left(R_{t}\right)\times t_{n}$$

where $E_{tx}\left(R_{t}\right)$ denotes the communication energy consumption of one information package transmitting. The movement energy consumption $M_{e}$ can be defined as the product of the movement distance $m_{d}$ and the energy consumption per movement distance $e_{mu}$, which can be also described as follows:

(6) $$M_{e}=m_{d}\times e_{mu}$$

(3) Node sensing model

The Boolean sensing model in [13] is adopted to describe node sensing. The function $f\left(p_{i},s_{i}\right)$ denotes whether the cube point $p_{i}$ can be covered by the node $s_{i}$:

(7) $$f(p_{i},s_{i})=\{\begin{array}{c} 1\;\left(\sqrt{{\left(x_{i}-a_{i}\right)}^{2}+{\left(y_{i}-b_{i}\right)}^{2}+{\left(z_{i}-c_{i}\right)}^{2}}\leq R_{s}\right)\\ 0\;\left(\sqrt{{\left(x_{i}-a_{i}\right)}^{2}+{\left(y_{i}-b_{i}\right)}^{2}+{\left(z_{i}-c_{i}\right)}^{2}}>R_{s}\right) \end{array}$$

where $\left(x_{i},y_{i},z_{i}\right)$ is the coordinate of the node $s_{i}$, $\left(a_{i},b_{i},c_{i}\right)$ is the coordinate of the cube point $p_{i}$, and $R_{s}$ is the sensing range of the node $s_{i}$. If the value of $f\left(p_{i},s_{i}\right)$ is 1, the cube point $p_{i}$ is covered by the node $s_{i}$. Otherwise, the cube point $p_{i}$ is not covered by the node $s_{i}$. Based on function $f\left(p_{i},s_{i}\right)$, the coverage degree $k\left(p_{i}\right)$ of the cube point $p_{i}$ can be defined as:

(8) $$k\left(p_{i}\right)=\sum_{j=1}^{n}f\left(p_{i},s_{j}\right)$$

where n denotes the total number of nodes in the network. Based on the coverage degree $k\left(p_{i}\right)$, the function $f_{0}\left(p_{i}\right)$ describes whether the cube point $p_{i}$ is covered or not:

(9) $$f_{0}\left(p_{i}\right)=\{\begin{array}{c} 1\;k\left(p_{i}\right)=0\\ 0\;k\left(p_{i}\right)\neq 0 \end{array}$$

If the value of $f_{0}\left(p_{i}\right)$ is 1, the cube point $p_{i}$ is not covered by any sensor.

### 3.2. Definitions

(1) Network coverage rate

The network coverage rate $C_{v}$ can be defined as the ratio of $p_{c}$ and $p_{t}$, where $p_{c}$ is the number of the cube points covered and $p_{t}$ is the total number of all the cube points. Therefore, $C_{v}$ can be calculated as follows:

(10) $$C_{v}=\frac{p_{c}}{p_{t}}$$

(2) Network connectivity rate

The network connectivity rate $C_{n}$ can be defined as the ratio of $n_{c}$ and n, where $n_{c}$ is the number of nodes that can communicate with the sink node through single-hop or multi-hop communication. $C_{n}$ can be calculated as follows:

(11) $$C_{n}=\frac{n_{c}}{n}$$

If the network connectivity rate is 1, the network achieves full network connectivity, and all the nodes can communicate with the sink node through single-hop or multi-hop communication.

### 3.3. Preliminaries

(1) Inspired by the similar assumptions in [12], the sink node and all the other nodes can freely move in all directions and their real-time locations can be known during the deployment process.

(2) Before the deployment, the destination location information of the sink node, *i.e.*, the location information of the center of the water surface, has been stored in the memory of all the other nodes for them to gain information on the destination location of the sink node. Information on the 3D underwater space model and the number of nodes has also been stored in the memory of the sink node. 

(3) The communication range of the sink node is $R_{t}$ and the sink node can be recharged, whereas the sensing capability of the sink node is neglected. All the other nodes are homogeneous, meaning these nodes have the same sensing range $R_{s}$, same communication range $R_{t}$, and initial energy $E_{i}$. Furthermore, each of them has a unique ID number.

## 4. Problem and Algorithm Description

The communication medium of UWSNs is acoustic signal and their network structure is 3D, but their node energy is limited and difficult to compensate. When UWSNs are applied to detect underwater disaster or underwater military actions or to finish other similar tasks, the system must have excellent real-time monitoring. All the nodes must be able to communicate with the sink node to guarantee full network connectivity. The deployment for UWSNs involves optimization to adjust the locations of nodes to improve the network coverage and minimize energy consumption of nodes during the process, with full consideration of the characteristics and requirements mentioned above.

Li *et al.* [14] modified the traditional 2D virtual forces algorithm [15] and applied it to the 3D case and then proposed the TVFD algorithm. They suggested that the locations of nodes must be adjusted for a few rounds. In each round, all the nodes broadcast their location information to their neighbor nodes in the communication range. These nodes can then calculate the total virtual force, which is the vector sum of the four kinds of virtual forces exerted on them, which are as follows: the traditional attraction virtual forces, traditional repelling virtual forces, central attraction force, and the localized equilibrium forces. Afterward, a node moves according to the total virtual force. This algorithm is a typical deployment algorithm for UWSNs comprising freely mobile nodes, and can improve the network coverage and connectivity rates. However, some improvements are still worthy of note based on the following aspects:

(1) The traditional attraction virtual forces contribute to the improvement of the network connectivity in the TVFD algorithm. However, node movement is determined by the total virtual force, and the traditional attraction virtual forces cannot completely control the movement of a node. Thus, the network cannot achieve full connectivity.

(2) The TVFD algorithm is operated in a distributed manner. In each round, any node can only move to a local optimized location instead of to a global optimized location. Thus, this kind of movement can only play a limited role in improving network connectivity.

(3) The TVFD algorithm is operated round by round. In each round, nodes are not only required to broadcast their location information but also to move according to the total virtual force exerted on them. To improve the network coverage rate and network connectivity rate, the algorithm needs to be operated for a few rounds, enlarging the communication and movement energy consumption.

We propose the DBCDS algorithm to provide a better solution to the deployment problem of UWSNs. After nodes are randomly scattered in the 3D monitoring underwater space, those disconnected to the sink node move toward the sink node until full network connectivity is achieved. The sink node then performs centralized optimization to determine the CDS of the network and continuously adjusts the locations of the dominated nodes. The dominated nodes are required to move toward an optimized location, which is in the communication range of the dominating nodes. A dominated node can improve the network coverage rate upon reaching the optimized location.

The two main parts of the proposed DBCDS algorithm are determining the CDS of the network and performing centralized optimization adjustment for the dominated nodes. A brief description of the whole process for the DBCDS algorithm is also provided.

### 4.1. CDS Determination

The CDS plays an important role in the virtual backbone construction of wireless sensor networks [31]. The dominating set is a subset of the set consisting of all the nodes of the networks. A node which does not belong to the subset is adjacent to at least one of the nodes in the subset. The node that belongs to the subset is called the dominating node, and the node that does not belong to the subset is called the dominated node. If the nodes in the dominating set are connected with another, the dominating set is called CDS [32], and the node that belongs to the dominating set is called the connected dominating node. For a network that has achieved full network connectivity, we first determine the CDS and then adjust the locations of the dominated nodes. If the new locations of the dominated nodes are still in the communication range of dominating nodes, the network can still keep full connectivity. This is the basic idea of DBCDS algorithm. After nodes are randomly scattered in the 3D monitoring underwater space, nodes disconnected to the sink node are required to move toward the sink node until full network connectivity is achieved. The sink node then performs centralized optimization to determine the CDS of the network and continuously adjusts the locations of the dominated nodes. The process wherein the sink node determines the CDS of the network is as follows:

(1) The sink node determines itself as the first paramount connected dominating node, and then it judges other nodes in the order of the $I_{d}$ number.

(2) The first round of judgment starts by initializing i=1, where i is the temporary number to label the node during the traversal of nodes.

(3) The sink node judges whether the node whose $I_{d}$ number is i has any two neighbor nodes nonadjacent to each other. The term “neighbor” here means distance between the node i and the other node, which is not bigger than the communication range. If such neighbors are present, the node i is determined as a primary connected dominating node. Otherwise, it is determined as a dominated node.

(4) Considering i=i+1, if i is bigger than n (*i.e.*, the total number of nodes), the first round of judgement ends. Otherwise, (3) is repeated.

(5) The second round of judgement starts by initializing i=1.

(6) All the neighbor nodes of the node whose $I_{d}$ number is i are considered, noting that the node i itself is also included in its neighbor nodes. Then, the sink node judges whether all the neighbor nodes are the primary connected dominating nodes. If it is, the node i is determined as a dominated node. Otherwise, it is a paramount connected dominating node.

(7) Considering i=i+1, if i is bigger than n (*i.e.*, the total number of nodes), the second round of judgement ends. Otherwise, (6) is repeated.

(8) The subset consisting of the paramount connected dominating nodes (including the sink node) is the CDS. The paramount connected dominating node can also be called the dominating node for short in this study. 

**Figure 2:**
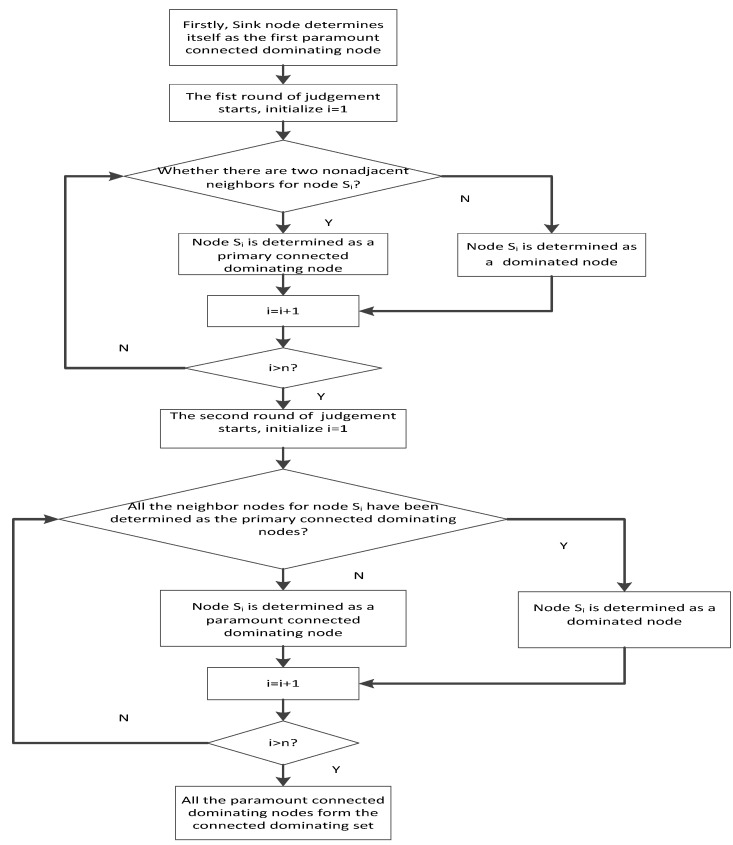
Process of CDS determination.

The above process is described in a flow chart in Figure 2.

### 4.2. Centralized Optimization Adjustment for Locations of Dominated Nodes

After determining the CDS, the sink node can perform central optimization to adjust the locations of the dominated nodes and can dispatch the dominated node to an optimized location, which is in the communication range of the dominating nodes. The dominated node can improve the network coverage rate upon reaching the optimized location.

The process wherein the sink node adjusts the locations of the dominated nodes is as follows:

(1) The adjustment round $r_{n}$ is initialized to be 0.

(2) The sink node calculates the network coverage rate before this adjustment round and then finds the max coverage blind cube point $B_{\max}$ using the following formula:

(12) $$\{\begin{array}{c} B_{\max}=\underset{p_{i}\in G}{\max}\;\left\{\sum_{p_{d}\in M}f_{0}(p_{d})\right\}\\ M=\left\{p_{d}|\sqrt{{\left(a_{d}-a_{i}\right)}^{2}+{\left(b_{d}-b_{i}\right)}^{2}+{\left(c_{d}-c_{i}\right)}^{2}}\leq R_{s}\right\} \end{array}$$

where G denotes the set that consists of the cube point whose distance to the dominating nodes is smaller than the communication range $R_{t}$. Mdenotes the set of the neighbor cube points of the cube point $P_{i}$ in G. Notably, “neighbor” here means the distance between the cube point $P_{i}$ and the other cube point, which is not bigger than the sensing range.

(3) The sink node calculates and finds the least important dominated node using the following equation:

(13) $$S_{\min}=\underset{s_{k}\in S}{\min}\left\{C_{vb}-C_{-s_{k}}\right\}$$

where S denotes the set that consists of the dominated nodes, $C_{vb}$ denotes the coverage rate before the adjustment round, and $C_{-s_{k}}$ denotes the coverage rate supposing that the node $s_{k}$ has been deleted from the network.

(4) Supposing that the coverage rate is $C_{va}$, if the node $S_{\min}$ has been moved to the max coverage blind cube point $B_{\max}$, then $C_{va}$ is compared with $C_{vb}$. If $C_{va}$ is bigger than $C_{vb}$, the sink node requires node $S_{\min}$to finish the real movement, and node $S_{\min}$ moves to the max coverage blind cube point $B_{\max}$ along the straight line. The adjustment round $r_{n}=r_{n}+1$ is obtained and (2) is repeated. Otherwise, the total optimization adjustment for locations of dominated nodes ends.

**Figure 3:**
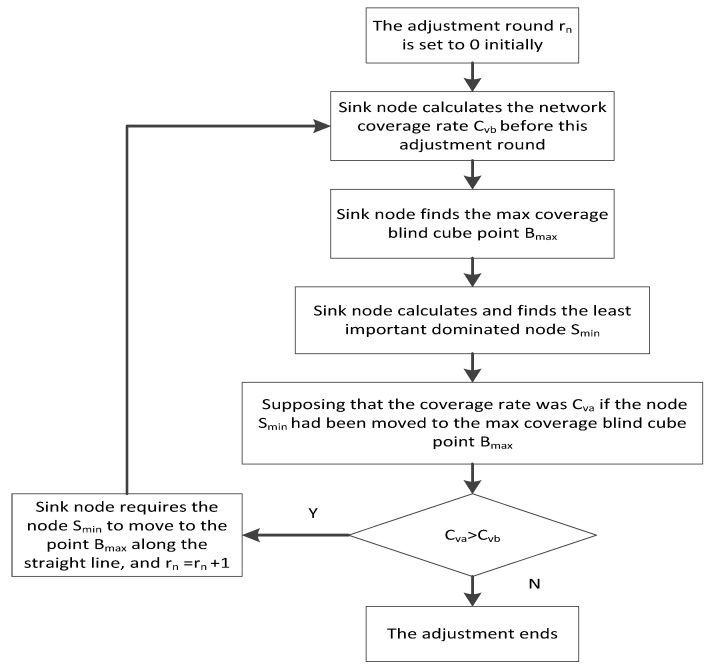
Adjustment process for the locations of dominated nodes by sink node.

The above process is described using the following flow chart in Figure 3.

For the 3D underwater space cube model, whether the small cube is covered or not depends on the cover state of its center point. Therefore, for the DBCDS algorithm, when the sink node performs central optimization to adjust the locations of the dominated nodes, the cube resolution *w* affects the accuracy and time complexity of the central optimization. If *w* is small, the accuracy and time complexity is high. For example, when the sink node finds the least important dominated node $S_{\min}$, for any dominated node $s_{k}$, the time complexities of calculating the $C_{vb}$ and $C_{-s_{k}}$ are $O(p_{t}\times n)$ and $O(p_{t}\times (n-1))$, respectively. The smaller *w* means the larger $p_{t}$, and then results in the higher time complexity.

### 4.3. Brief Description of DBCDS Process

After elaborating the two main parts of the DBCDS algorithm, *i.e.*, determining the CDS of the network and performing centralized optimization adjustment for the dominated nodes, a brief description of the entire process for the DBCDS algorithm is provided.

(1) All the nodes are randomly scattered on the surface of the 3D monitoring underwater space. Then, the sink node moves and is fixed at the center of the water surface. All the other nodes randomly adjust their own depths. 

(2) The sink node broadcasts the ready message. If a node receives the message, it continuously broadcasts the message and sends its location information to the sink node. Otherwise, a node that cannot receive the message has to move toward the sink node until it receives the message and then sends its location information to the sink node.

(3) If all the nodes receive the ready messages, the number of the location information received by the sink node is equal to the number of nodes. Thus, the sink node can judge whether the network achieves full connectivity or not from the relationship between the number of the location information received and the number of nodes. If they are not equal, the network is still not fully connected, and the sink node waits until the equality is achieved. After this, the network becomes fully connected and the process proceeds to (4).

(4) The sink node determines the CDS of the network.

(5) The sink node performs centralized optimization to adjust the locations of the dominated nodes.

(6) The DBCDS algorithm ends.

**Figure 4:**
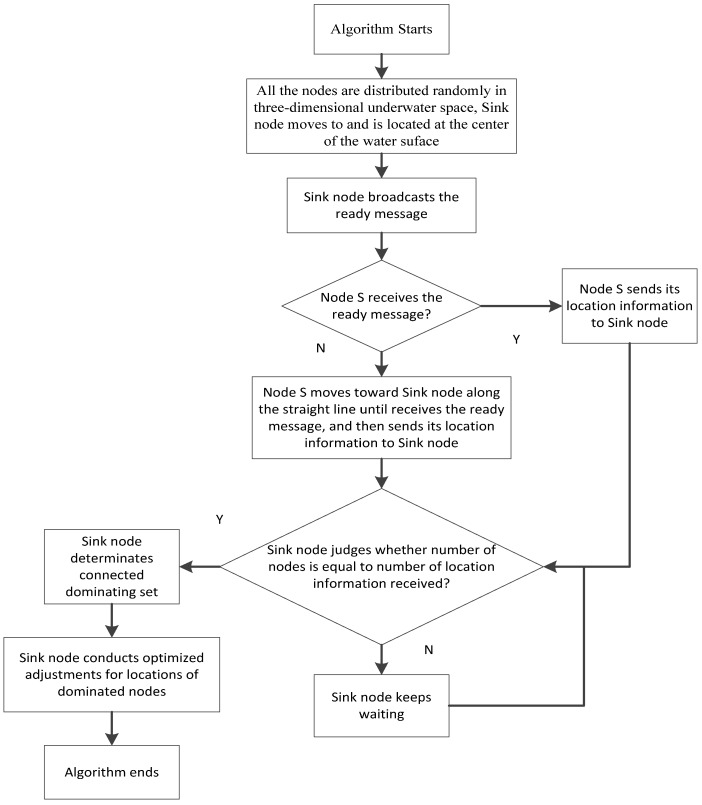
Process of DBCDS algorithm.

The abovementioned process is also described using the following flow chart in Figure 4.

## 5. Simulation Evaluation

### 5.1. Comparison Algorithm and Evaluation Metric

The TVFD algorithm is one of the typical deployment algorithms for UWSNs based on the assumption that nodes can freely move in all directions. Thus, this algorithm is chosen and compared with DBCDS algorithm based on the following evaluation metrics: network coverage rate, network connectivity rate, communication, and movement energy consumption during node deployment.

Compared with the TVFD algorithm, the DBCDS algorithm has several advantages, as follows:

(1) Nodes disconnected to the sink node are required to move toward the sink node until full network connectivity is achieved. Only the locations of the dominated nodes are adjusted, and the new locations of the dominated nodes are in the communication range of the dominating nodes. Thus, full network connectivity can still be maintained after the adjustment. 

(2) The locations of the dominated nodes in the above adjustment are determined by the sink node through centralized optimization, thereby creating larger network coverage rate improvement.

(3) The communication times between nodes are little, and movement nodes are usually disconnected nodes and dominated nodes. Therefore, the movement distance of the nodes dramatically decreases. The communication and movement energy consumption can then be effectively decreased.

### 5.2. Simulation Scenario and Parameter Settings

The MATLAB software is used to simulate the algorithms. The final results shown in the following figures are the average of the 50 simulations to eliminate the effect of simulation randomness. The length and width in the horizontal direction of the simulative 3D monitoring underwater space are the same at 120 m, whereas the depth of the space is 60 m. The cube resolution *w* is 5 m. To improve the network coverage rate and the network connectivity rate in the TVFD algorithm, the number of its running rounds is set to 20. Other main parameter settings of these two algorithms are enumerated in Table 1.

### 5.3. Simulation Results and Analyses

**Figure 5:**
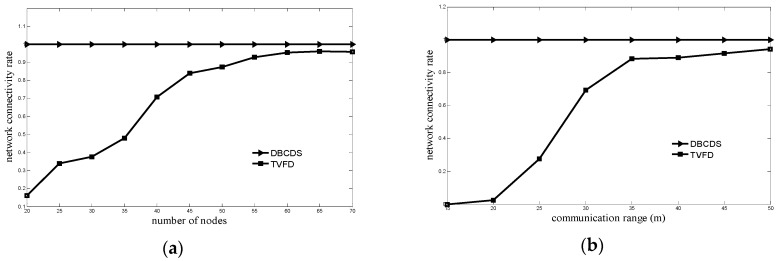
Comparison of network connectivity rate: (**a**) Comparison of network connectivity rate when number of nodes varies; (**b**) Comparison of network connectivity rate when communication range varies.

Figure 5 shows the comparison between the network connectivity rate of the TVFD and DBDCDS algorithms. As shown in Figure 5a, the simulation scenario is that the number of nodes is varying and the communication range of the node is set at 30 m, whereas Figure 5b shows a simulation scenario that the communication range of the node is varying and the number of nodes is set at 40 m. Based on Figure 5, if the number or communication range of the node is the same with that in the TVFD algorithm, the DBCDS algorithm can achieve full connectivity (*i.e.*, the network connectivity rate is 1). The traditional attraction virtual forces contribute to the improvement of the network connectivity rate in the TVFD algorithm. However, node movement is determined by the total virtual force, and the traditional attraction virtual forces cannot completely control the movement of a node. Thus, the network cannot achieve full connectivity. For the DBCDS algorithm, nodes disconnected to the sink node are required to move toward the sink node until full network connectivity is achieved. After determining the CDS of the network, the optimized adjustment of the locations of the dominated nodes is under the condition that the dominated nodes are ensured to be in the communication range of the dominating nodes. Therefore, full network connectivity can still be maintained after the adjustment.

**Figure 6:**
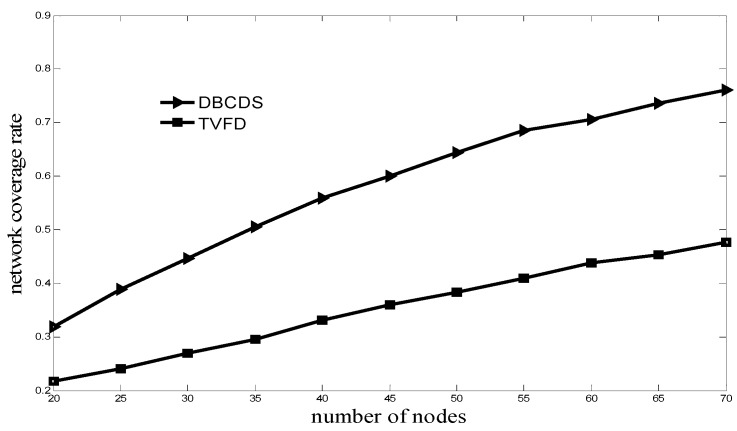
Comparison of network coverage rate.

Figure 6 shows the comparison of the relationship between the network coverage rate and the number of nodes for the TVFD and DBCDS algorithms, which can achieve higher network coverage rate than the former if the number of nodes is the same. In the TVFD algorithm, node movement is determined by the total virtual force produced by its neighbor nodes in the local area. This movement can only somewhat improve the network coverage rate in a local way. However, for the DBCDS algorithm, after the sink node determines the CDS of the network, it adjusts the locations of the dominated nodes in an optimized and global way, thereby achieving a larger improvement in the network coverage rate.

**Figure 7:**
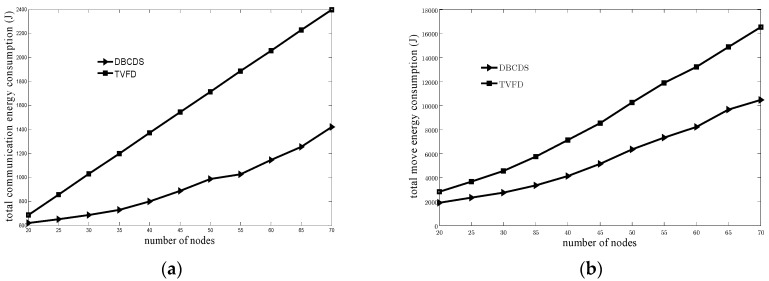
Comparison of total energy consumption: (**a**) Comparison of total communication energy consumption with various numbers of nodes; (**b**) Comparison of total movement energy consumption with various numbers of nodes.

Figure 7 shows the comparison of relationship between the total energy consumption of nodes and the number of nodes for the TVFD and DBCDS algorithms. Figure 7a shows the comparison of relationship between the total communication consumption of nodes and the number of nodes, whereas Figure 7b shows the comparison of relationship between the total movement consumption of nodes and the number of nodes. Based on Figure 7a, the total communication consumption of nodes for the TVFD algorithm exhibits a linear relationship with the number of nodes and is bigger than that for the DBCDS algorithm if the number of nodes is the same. The reason is that, for the sake of improving the network coverage rate and the network connectivity rate, the TVFD algorithm has to be run for several rounds, and in each round, all the nodes have to broadcast their location information once. Given that the running round for the algorithm is set at the same value (*i.e.*, 20 in the simulation) on the condition of different numbers of nodes, the communication consumption for each node is the same, that is, the amount of the energy consumption for transmitting the information package 20 times. Therefore, the total communication consumption of nodes has a linear relationship with the number of nodes and is relatively large. The total communication consumption of nodes in the DBCDS algorithm is relatively smaller because its optimization calculation is finished by the sink node and its main communication energy consumption of nodes only lies on three processes, namely, broadcasting the ready messages from the sink node to all other nodes, spreading information on the location information from all the other nodes to the sink node, and optimizing the adjustment for the dominated nodes by the sink node. As shown in Figure 7b, if the number of nodes is the same, the total movement energy consumption of nodes for the DBCDS algorithm is smaller than that for the TVFD algorithm. After all the nodes in the TVFD algorithm initially and randomly move in a vertical direction, each of them has to move according to the total virtual force exerted on them. Therefore, the total movement distance and total movement energy consumption of nodes are relatively larger. However, in the case of DBCDS algorithm, only a few nodes disconnected from the sink node move toward the sink node. After full network connectivity is achieved, only the locations of some dominated nodes are adjusted by the sink node in an optimized way. Therefore, the total movement distance and energy consumption of nodes are relatively smaller.

## 6. Conclusions

In this study, we propose a node DBCDS algorithm for UWSNs consisting of freely mobile nodes for the first time to solve the problem in which existing deployment algorithms for UWSNs cannot improve the network coverage rate under the premise of ensuring full network connectivity and do not optimize the communication and movement energy consumption during deployment. After nodes are randomly scattered in the 3D monitoring underwater space, nodes disconnected to the sink node are required to move toward the sink node until full network connectivity is achieved. The sink node then performs centralized optimization to determine the CDS of the network and continuously adjusts the locations of dominated nodes. The simulation results show that the proposed DBCDS algorithm can achieve a high coverage rate under the premise of ensuring the full connectivity, as well as decrease the communication and movement energy consumption during deployment compared with the typical deployment algorithm for UWSNs consisting of freely mobile nodes, *i.e.*, the TVFD algorithm. Considering the node drift or dying in real underwater environment, how to solve the problem of network topology adjustment during the network operation is one of our future research directions. Since the node deployment problem involves ensuring the full network connectivity, maximizing the network coverage rate, and minimizing the communication and movement energy consumption making it a multi-objective optimal problem, how to get an optimal or near-optimal solution to such a problem in a centralized way also deserves our future research.

## Figures and Tables

**Table 1:** Parameter Settings.

Parameter Names	Parameter Values
Initial energy of node (*E_i_*)	10,000 J
Power threshold (*P_r_*)	0.05 W
Size of information package (*M_b_*)	1 Kbit
Transmission speed of information package (*S_v_*)	5 Kbps
Energy spreading factor (*λ*)	1.5
Frequency of carrier acoustic signal (*f*)	25 kHz
Energy consumption per move distance (*e*_mu_)	1.5 J/m
Sensing range of node (*R_s_*)	15 m
